# 3D printing materials and 3D printed surgical devices in oral and maxillofacial surgery: design, workflow and effectiveness

**DOI:** 10.1093/rb/rbae066

**Published:** 2024-06-27

**Authors:** Xiaoxiao Wang, Min Mu, Jiazhen Yan, Bo Han, Rui Ye, Gang Guo

**Affiliations:** State Key Laboratory of Oral Diseases & National Center for Stomatology & National Clinical Research Center for Oral Diseases & Department of Orthodontics, West China Hospital of Stomatology, Sichuan University, Chengdu, Sichuan 610041, China; Department of Biotherapy, State Key Laboratory of Biotherapy and Cancer Center, West China Hospital, Sichuan University, Chengdu, Sichuan 610041, China; Department of Biotherapy, State Key Laboratory of Biotherapy and Cancer Center, West China Hospital, Sichuan University, Chengdu, Sichuan 610041, China; School of Mechanical Engineering, Sichuan University, Chengdu, Sichuan 610065, China; School of Pharmacy, Shihezi University, and Key Laboratory of Xinjiang Phytomedicine Resource and Utilization, Ministry of Education, Shihezi, 832002, China, Shihezi 832002, China; State Key Laboratory of Oral Diseases & National Center for Stomatology & National Clinical Research Center for Oral Diseases & Department of Orthodontics, West China Hospital of Stomatology, Sichuan University, Chengdu, Sichuan 610041, China; Department of Biotherapy, State Key Laboratory of Biotherapy and Cancer Center, West China Hospital, Sichuan University, Chengdu, Sichuan 610041, China

**Keywords:** 3D printing, oral and maxillofacial surgery, patient-specific implants, surgical guides, splints

## Abstract

Oral and maxillofacial surgery is a specialized surgical field devoted to diagnosing and managing conditions affecting the oral cavity, jaws, face and related structures. In recent years, the integration of 3D printing technology has revolutionized this field, offering a range of innovative surgical devices such as patient-specific implants, surgical guides, splints, bone models and regenerative scaffolds. In this comprehensive review, we primarily focus on examining the utility of 3D-printed surgical devices in the context of oral and maxillofacial surgery and evaluating their efficiency. Initially, we provide an insightful overview of commonly utilized 3D-printed surgical devices, discussing their innovations and clinical applications. Recognizing the pivotal role of materials, we give consideration to suitable biomaterials and printing technology of each device, while also introducing the emerging fields of regenerative scaffolds and bioprinting. Furthermore, we delve into the transformative impact of 3D-printed surgical devices within specific subdivisions of oral and maxillofacial surgery, placing particular emphasis on their rejuvenating effects in bone reconstruction, orthognathic surgery, temporomandibular joint treatment and other applications. Additionally, we elucidate how the integration of 3D printing technology has reshaped clinical workflows and influenced treatment outcomes in oral and maxillofacial surgery, providing updates on advancements in ensuring accuracy and cost-effectiveness in 3D printing-based procedures.

## Graphical Abstract

**Figure:**
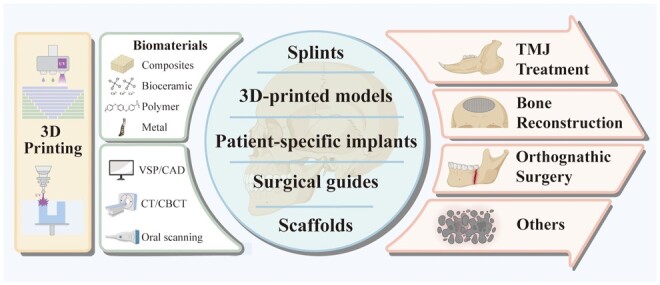

## Introduction

Oral and maxillofacial surgery is a speciality devoted to the diagnosis and management of conditions affecting the oral cavity, jaws, face and related structures. While this field offers considerable advancements and benefits, it presents several challenges that surgeons must navigate, including notable anatomical diversity among individuals, surgical complexity and technological limitations [1, 2]. 3D printing, also known as additive manufacturing, presents as an innovative technology that enables the production of three-dimensional objects from digital designs [3]. Departing from conventional manufacturing methods, which often involve subtractive processes such as cutting or drilling materials [4], 3D printing constructs objects layer by layer. This approach allows for the fabrication of intricate and complex geometries that would be difficult or unfeasible to achieve using traditional methods [5, 6].

**Figure 1.**
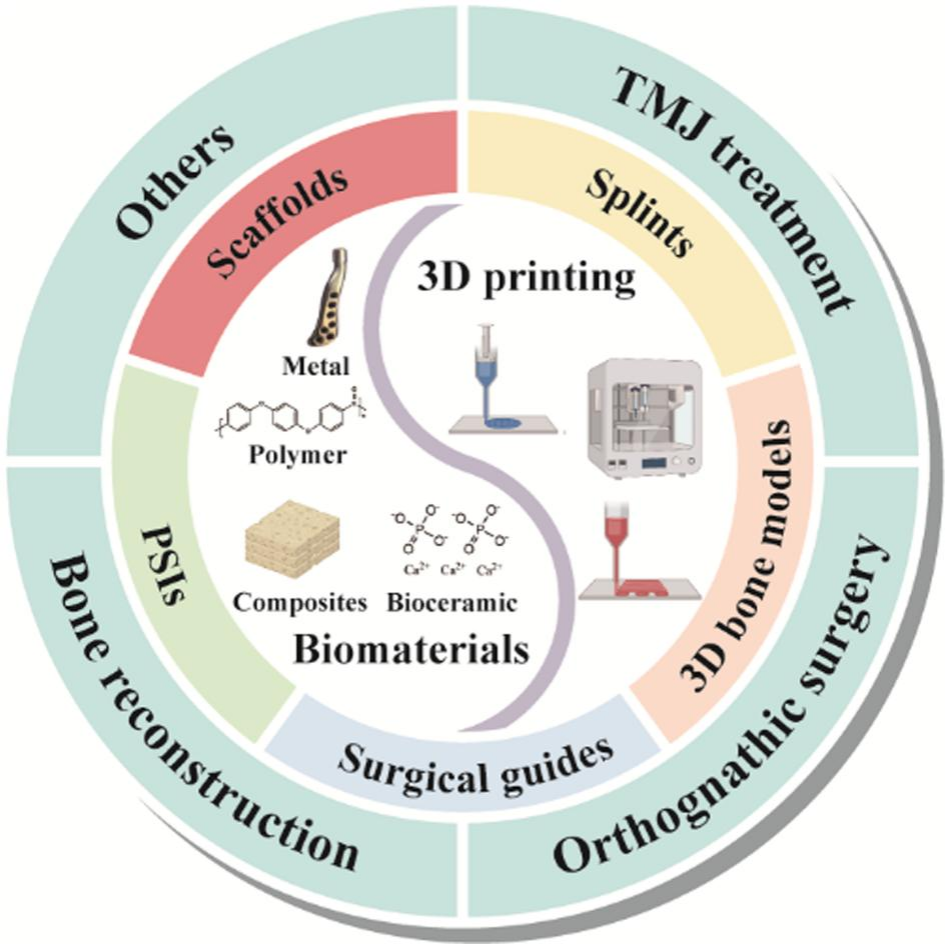
Schematic of 3D printing and 3D-printed surgical devices in oral and maxillofacial surgery. This figure was created using BioRender (https://biorender.com/).

A pivotal advantage of 3D printing lies in its ability to create intricate structures and customized items with exceptional precision [7, 8]. This capability empowers designers and surgeons with unprecedented flexibility to optimize designs and create patient-specific implants (PSIs) and complementary surgical tools tailored to possess high functionality [9, 10]. Consequently, the applications of 3D-printed PSIs, surgical guides, splints regenerative 3D models and regenerative scaffolds have extended across diverse domains, including bone reconstruction in oral and maxillofacial regions (OMFs), orthognathic surgery and temporomandibular joint (TMJ) treatment and other applications (Figure 1). By conducting digital design on patient-derived anatomical data, obtained via techniques such as computed tomography (CT) scans or intraoral scanning, surgeons can develop implants, surgical guides and other implements precisely aligned with the patient’s anatomy. This precision ensures the accuracy of surgical interventions, improves patient outcomes and reduces complication rates [11]. Moreover, by generating 3D-printed replicas of the patient’s anatomical structure, surgeons gain better insights into pathological anatomy and can simulate surgical plans, enabling preoperative analysis, assessment of diverse treatment methodologies and anticipation of potential challenges or complications. This preoperative preparation enhances efficiency and efficacy in surgical planning, ultimately leading to improved patient care [12, 13].

Additionally, 3D printing demonstrates the potential to optimize production procedures by eliminating the need for manual fabrication or adjustments of implants and surgical guides. This increased efficiency translates to shorter timeframes, shorter surgical waiting times and augmented overall operational efficiency within the operating room [14]. As a result, 3D printing has become an indispensable tool for innovation and problem-solving across a wide range of challenges encountered in oral and maxillofacial surgery. Therefore, this review primarily focuses on examining the utility of 3D-printed surgical devices in the context of oral and maxillofacial surgery and evaluating their efficiency. Initially, we provide an overview of commonly used 3D-printed surgical devices, discussing their innovation, clinical applications and the materials and technologies involved. Additionally, we delve into the rejuvenating impact of 3D-printed surgical devices within various oral and maxillofacial surgery subdivisions, with particular emphasis on bone reconstruction, orthognathic surgery and TMJ treatment. We discuss how 3D printing has reshaped clinical workflow and treatment outcomes in oral and maxillofacial surgery while updating the progress in enhancing accuracy in 3D printing-based procedures.

## Common surgical devices and suited materials established for oral and maxillofacial surgery

**Figure 2.**
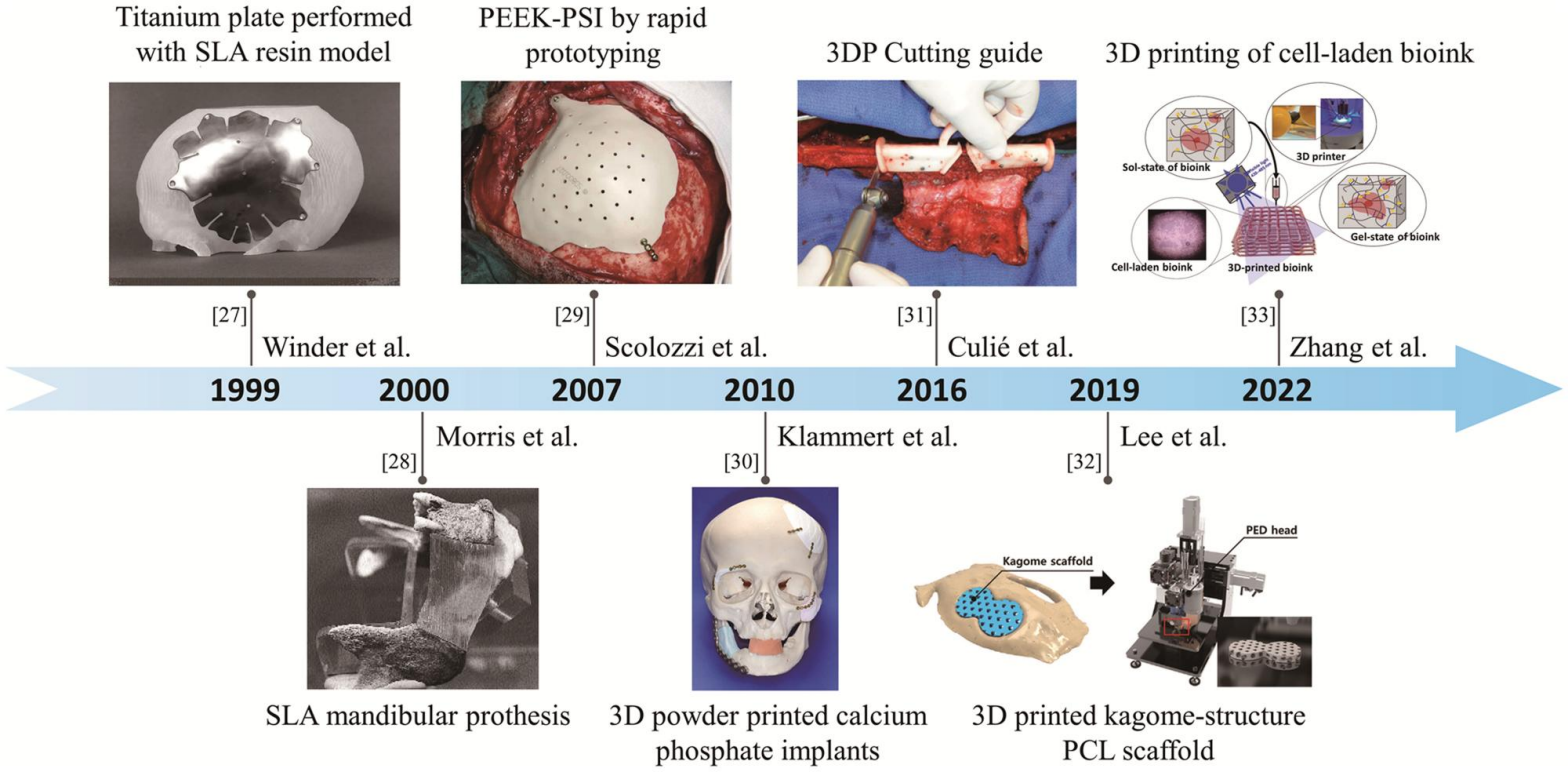
Timeline of applications of 3D printing and 3D printed surgical devices in oral and maxillofacial surgery. Custom titanium plate performed with stereolithography resin model for cranioplasty in 1999 [27] (from Ref. [27] licensed under Taylor & Francis license). Stereolithography appearance (SLA) mandibular prosthesis fitted within the mandibular defect site in 2000 [28] (from Ref. [28] licensed under John Wiley & Sons license). Polyetheretherketone (PEEK)-PSI by rapid prototyping for orbito-fronto-temporal reconstruction in 2007 [29] (from Ref. [29] licensed under Wolters Kluwer license). 3D powder printed calcium phosphate (CP) implants for craniofacial defects reconstruction in 2010 [30] (from Ref. [30] licensed under Elsevier license). 3DP cutting guide for fibular free-flap harvesting in 2016 [31] (from Ref. [31] licensed under Elsevier license). 3D-printed kagome-structure scaffold for bone regeneration in 2019 [32] (from Ref. [32] licensed under Elsevier license). 3D-printed cell-laden light-curable chitosan scaffold for bone tissue regeneration in 2022 [33] (from Ref. [33] licensed under Elsevier license).

**Figure 3.**
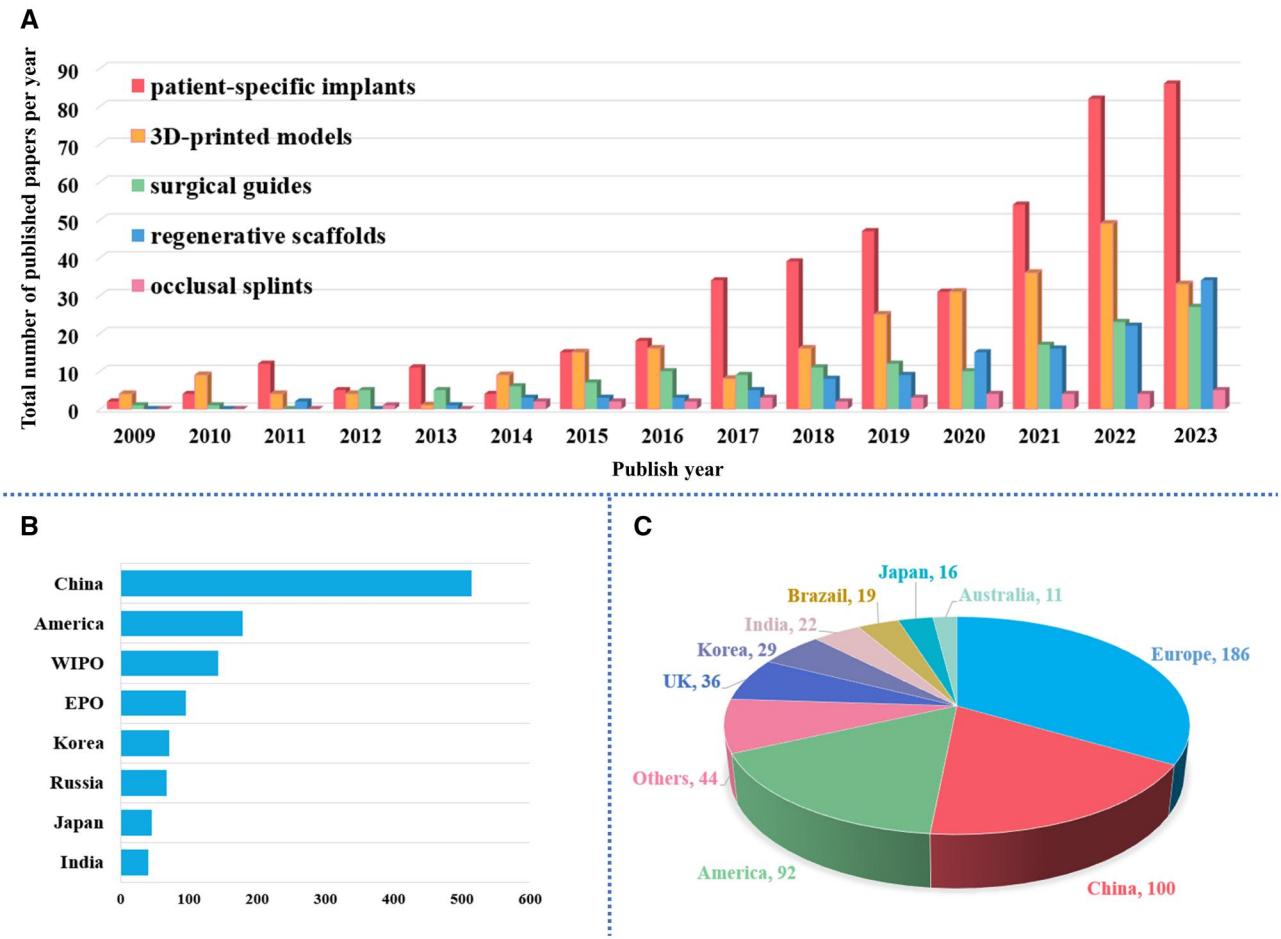
Statistic graphics of papers and patent status of 3D-printed surgical devices in oral and maxillofacial surgery in recent 15 years. (**A**) Publication status of papers on 3D-printed surgical devices in oral and maxillofacial surgery in the past 15 years. (**B**) The patent application status in each area of the world. (**C**) The total number of Investigator-Initiated Clinical Trial/Research (IIT/IIR) and Industry-Sponsored Clinical Trial (IST) in each area of the world.

We created a table that provides detailed information on commonly utilized surgical devices, including data sources, software, materials, printers, applications and key features (see Table 1). Furthermore, we developed a timeline of applications of 3D printing and 3D-printed surgical devices in oral and maxillofacial surgery to visually illustrate significant milestones and innovations within the scope of our review (Figure 2). Additionally, we conducted database searches and generated statistical graphics to analyze the patent status and paper publications related to 3D-printed surgical devices in oral and maxillofacial surgery over the past 15 years (Figure 3). See methodology in Supplementary data.

**Table 1.** Commonly used surgical devices in oral and maxillofacial surgery with detailed information

3D-printed devices	Data source	Materials	Design software	3D printer	Printing technology	Applications	Key features	Ref
Surgical guides	CBCT & intraoral scan	Ti6Al4V ELI	Magics	Metalsys15	SLM	Orthognathic surgery: Le Fort 1 osteotomy and BSSO	Reduced time: mean operation time is 5.76 ± 0.43 h. Accuracy and stability: the mean difference in accuracy is 0.485 ± 0.280 mm.	[15]
	CT	Polyamide	3-Matic and Magics	NM	NM	Mandible and donor osteotomy guides	In 18 cases, the difference between planned and actual reconstruction was minimal. Function improved postoperatively.	[11]
	CT	Resin	3-Matic	Objet Eden260VS	PolyJet	Cutting and repositioning templates and final splint for maxilla repositioning in bimaxillary orthognathic surgery	Accuracy: the mean linear distance between the planned and actual postoperative position is 1.17 ± 0.66 mm.	[16]
	CT	MED610 resin or NextDent SG	3-Matic 13.0	NM	NM	Patient-specific fibula malleolus cap for harvesting	Significantly reduced deviations in locations and angles of distal fibula osteotomies. Significantly increased accuracy of implant platform locations.	[17]
Patient-specific implant	CT/MRI	PEEK	Mimics	Intamsys FUNMAT HT	FFF	Reconstruction of zygomatic deformities	Great load resistance and safety under heavy loading: maximum stress of approximately 0.89 MPa, strain of about 2.2 × 10^−4^, deformation at about 14 μm. Fitting accuracy: a maximum deviation of 0.4810 mm.	[18]
	CBCT	Glass-ceramic (BGS-7)	3-Matic	NM	NM	Reconstruction of zygomatic bone defects	Implant-bone fusion: the average implant-bone fusion rate was 76.97% at 6 months after surgery. Accuracy: the average displacement distance of all 10 implants was 0.4149 mm.	[19]
	CT	PC-ISO	Mimics	Stratasys FDM^®^Fortus 900mc	FDM	Orbital fractures restoration	Minimal cost and less production time. Regain of reasonable orbital floor and better facial appearance.	[20]
	CT	Titanium MG1	CAD	AM250 3D metal printer	Laser fusion	Custom-made bone-anchored titanium prosthesis for reconstruction after total bilateral maxillectomy	Total cost: €8000 and 7 weeks. Good functional and anatomical outcomes.	[21]
	NM	PCL/β-TCP mixture	3-Matic	Multi-head deposition system	NM	PCL/β-TCP scaffolds for mandibular reconstruction	Significant higher bone volume in Micro-CT. More bone formation in histomorphometric analysis.	[22]
Splints	Cast scanning	Formlabs Clear Resin	Dolphin	Form 2	SLA	Single-jaw orthognathic surgery	Fabrication time: 5∼9 h. Average cost: 0.73 Canadian dollars.	[23]
	CT and cast scanning	Resin	Geomagic Freeform	ProJet 7000	SLA	Orthognathic surgery	Proposed a complete digital workflow. Accuracy: satisfactory translational (linear) and rotational (angular) discrepancies.	[24]
	Resin models scanning	Resin	Geomagic Studio 2013	NOVA3D Bene3	SLA	Orthognathic surgery	A reasonable offset improves the precision of 3D-printed splints. ISs and FSs with both 2-mm and 3-mm OCD are clinically beneficial.	[25, 26]

NM, not mentioned.

### Patient-specific cutting and positioning guides

**Figure 4.**
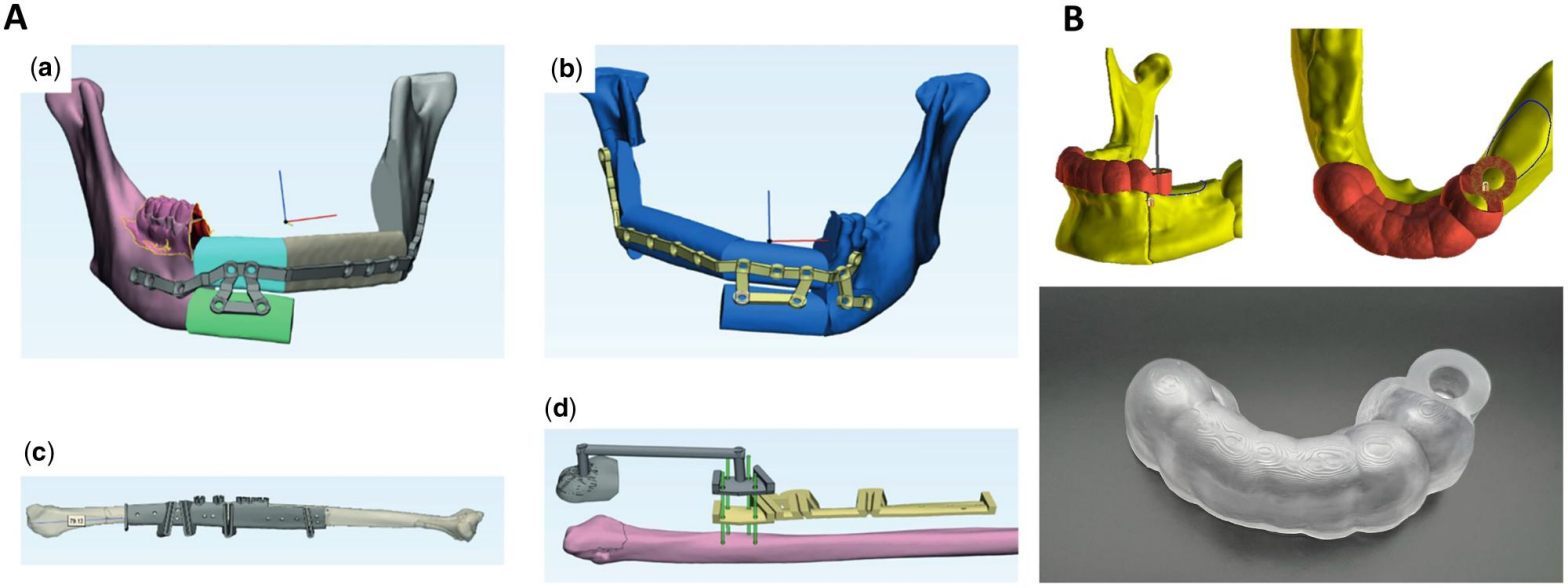
3D Printed surgical guides for precise cutting and positioning. (**A**) Conventional method vs. malleolus cap method for fibula flap harvest: (a) virtual planning in the control group; (b) virtual planning in the study group; (c) single fibula harvest guide in the control group; (d) two combinational guides with segmentation guide, fibula cap, and screw holes in the study group [17] (from Ref. [17] licensed under Creative Commons Attribution 4.0 license). (**B**) A virtual and 3D-printed surgical guide [39] (from Ref. [39] licensed under Elsevier license).

Throughout the excision of damaged areas, the integration of customized cutting guides can significantly assist in precisely determining the location and orientation of osteotomies, while repositioning guides can establish the optimal placement of bone grafts for prosthesis rehabilitation [11, 34]. These guides have demonstrated efficacy in enhancing accuracy, reducing operative time, facilitating complex reconstructions and aiding intraoperative decision-making in various procedures such as orthognathic osteotomies [15, 16], necrotic/tumorous bone resection [11], donor bone harvesting [11, 17, 35], bony segments repositioning [36], implants fitting [37] and bone biopsies [38]. For instance, Pu *et al.* [17] introduced a novel fibula malleolus cap for fibula flap harvesting to address challenges encountered in reducing the extent of deviations in terms of locations and angles of distal fibula osteotomies (Figure 4A).

### Splints

During a two-jaw surgery, intermediate splints (ISs) are used to align the maxilla with the mandible, followed by final splints (FSs) to relocate the mandible to acquire the final occlusion. Conventional plaster model-based surgery is associated with inherent inaccuracies originating from steps such as impression-taking, model pouring, trimming, mounting, face-bow transfer and manual splint fabrication. In contrast, virtual surgical planning and 3D printing have offered a more efficient workflow with digital splints, theoretically offering greater accuracy by eliminating these sources of deviation [40]. Nevertheless, printed splints might not always fit well within dentitions due to potential systematic errors that can occur during digital scanning and 3D printing procedures. Two studies innovatively proposed to fabricate splints from positively offset dental models, particularly focusing on interproximal areas that may not be clearly captured by dentition scanning, which could enhance the fit of splints [25, 41]. According to Wang *et al.* [26], it is recommended that both 2 or 3-mm occlusal coverage depth (OCD) for ISs and FSs can be considered due to their precision relative to clinical acceptability. After carefully conducted design of digital splints, resin materials are 3D-printed using commercial printers, such as ProJet 7000 [24], Objet Eden260VS [16], NOVA3D Bene3 [25], D20II [42] and others, utilizing stereolithography (SLA) or digital light processing (DLP) technology.

### Patient-specific implants

Standard-sized implants and autogenous bone grafts are considered as the gold standard and employed in conventional treatments to restore bone defects. However, these methods necessitate customization to fit the shape of the defects, which can be labor-intensive and time-consuming [43]. This challenge is particularly pronounced in oral and maxillofacial regions, where complex anatomical structures like the orbital floor pose difficulties in obtaining an exact 3D shape. Even minor discrepancies between the implant and the bone defect can lead to implant instability or failure. To address these challenges, 3D printing has emerged as a promising alternative for fabricating customized implants that precisely match the original structures in a shorter timeframe [44].

**Figure 5.**
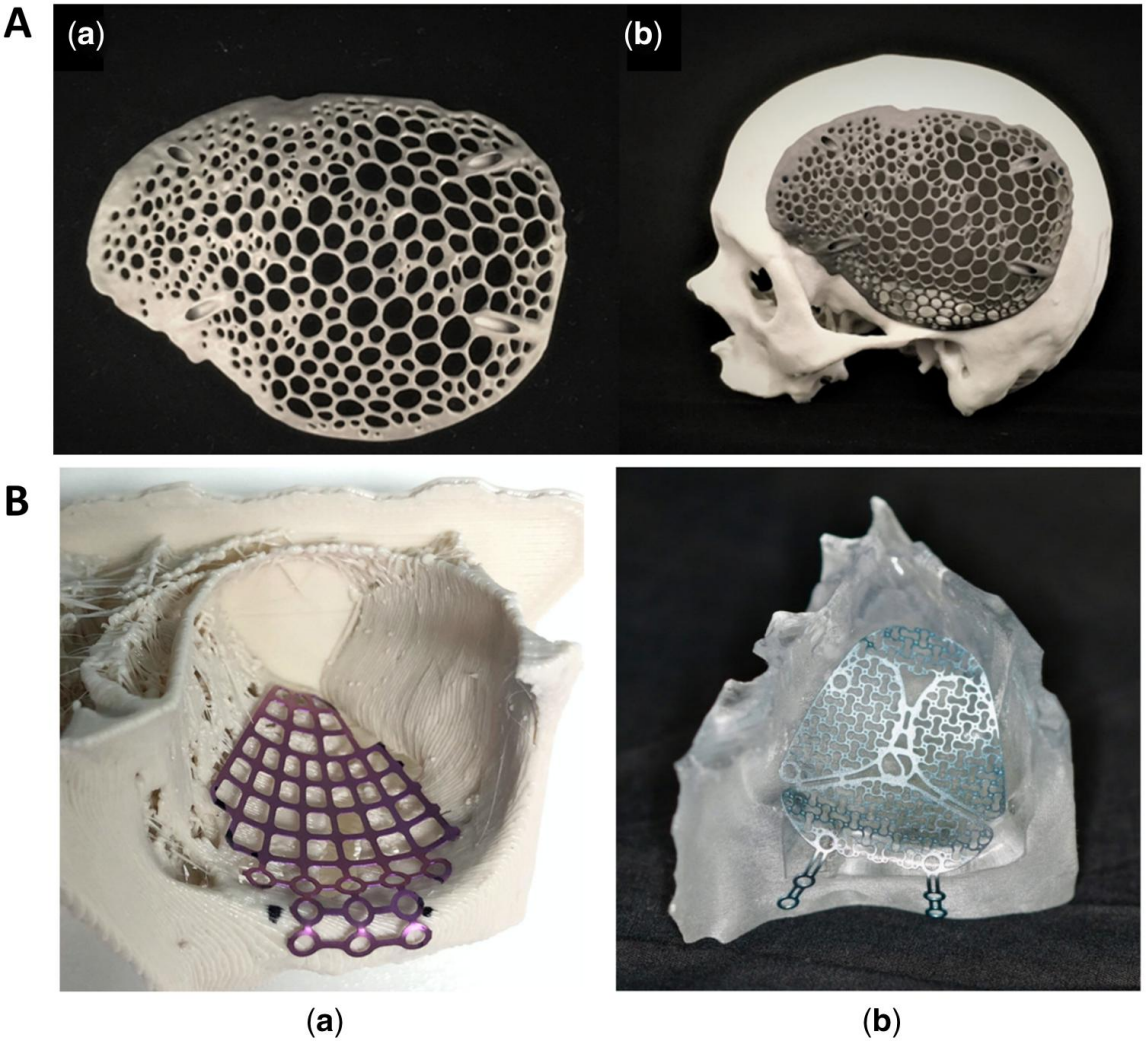
3D-printed PSI for defective bone reconstruction. (**A**) (a) Voronoi pattern cranial implant by SLM; (b) a skull biomodel demonstrating accurate fit (from Ref. [47] licensed under Creative Commons Attribution 4.0 license). (**B**) Orbital pre-bent plates made from a 3D printing anatomical model: (a) matrix MIDFACE mesh plate on a polylactic acid (PLA) model; (b) medartis modus midface OPS 1.5 plate on a clear MED610 model (from Ref. [52] licensed under Creative Commons Attribution 4.0 license).

Notably, 3D-printed titanium implants have garnered significant attention, particularly for reconstructing large-sized load-bearing areas. Titanium implants offer exceptional high strength-to-weight ratio, rigidness, biocompatibility, anti-infection, corrosion resistance, and nonmagnetic properties [36, 45]. However, they are often costly and heavier than the original anatomy, leading to subsidence resulting from stress shielding effects and disparities in elasticity modulus. [43]. To address these issues, researchers have explored the use of biocompatible polymer materials with reduced weight [20, 46]. Others have focused on optimizing the internal configuration of titanium implants using 3D printing methods such as selective laser melting (SLM) [47] and electron beam melting (EBM) [48]. These methods allow for the design of desired internal configurations, maintaining porosity and pore continuity to reduce implant weight and enhance osteointegration. Li *et al.* [49] designed Ti–6Al–4V plate with a honeycomb structure via SLM. After 6 months, the bone tissue has integrated with and enveloped the honeycomb scaffold, indicating significate now bone formation and effective integration with the scaffold and showing its potential clinical application. Furthermore, 3D printing enables the fabrication of biomimetic implants with complex internal structures resembling natural bones. Sharma *et al.* [47] designed and printed a biomimetic customized titanium cranial implant for cranioplasty, incorporating an interconnected strut macrostructure that mimics bone trabeculae, utilizing the voronoi diagram as a basis for its construction. This voronoi design, weighing only 30 g, needed less material and fabrication time while exhibiting excellent protective strength (Figure 5A). Additionally, surface modification techniques have been explored to enhance implant-bone fusion [48, 50]. Major *et al.* [51] proposed PSI with a metallic core (TiAlNb7 alloy) and a bioactive coating (Polylactide (PLA) granulate with β-tricalcium phosphate (β-TCP) addition) to stimulate bone growth.

To address these challenges, biodegradable/bioabsorbable polymer biomaterials, such as polycaprolactone (PCL), polylactic acid (PLA), polyetheretherketone (PEEK), polyvinyl chloride (PVC) and others have gained popularity due to their rigidity, lightweight nature and biocompatibility [52–55]. Polyaryletherketone polymers, such as PEEK, are particularly suitable for use as bone substitutes due to their high strength, superior chemical resistance, light weight, radiolucency, reduction of CT artifacts and similar elastic modulus to human bones [53, 56–58]. Bioceramics, such as β-TCP or hydroxyapatite (HA), have been confirmed to promote osteoblastic differentiation of human mesenchymal stem cells, promoting osteogenesis and improving bone-implant contact ratio [52, 59, 60]. Bioceramics are frequently employed as bone void fillers in reconstructive surgery. However, their mechanical brittleness and the inability to directly fabricate implants limit their application as personalized implants [61]. However, integrating bioceramics into biodegradable polymeric matrices to develop composite bone-promoting implants enhances their bioactivity and may endow osteoinductivity to the composite biomaterials [22, 62–65]. Guillaume *et al.* [2] proposed a novel HA-filled poly(trimethylene carbonate) (PTMC) implant for orbital floor repair, designed to provide temporary support to the orbital content and degrade over time while stimulating neo-bone formation. Additionally, Lee *et al.* [19] conducted a clinical trial to assess the efficacy and stability of the 3D-printed CaOSiO_2_-P_2_O_5_-B_2_O_3_ glass-ceramics (BGS-7) implants. Cone beam computed tomography (CBCT) analysis obtained after 6 months revealed 100% bone fusion and an average fusion rate of 76.97%. No osteolysis was observed around implants, and the mean displacement distance was 0.4149 mm for all 10 implants. The satisfaction score on the visual analog scale was 9, and no adverse events were observed in any of the cases.

### 3D model

Oral and maxillofacial surgeries present unique challenges including delicate anatomical structures and limited operative field exposure. Surgical simulation is thus becoming essential in medical training, offering a controlled setting for refining surgical skills [66, 67]. While cadaveric bone has conventionally been established as the gold standard for simulation, its limited availability and high costs pose significant obstacles to widespread adoption [1, 68]. Consequently, 3D-printed bone models have emerged as a cost-effective and readily accessible alternative, providing highly realistic training resources [69].

**Figure 6.**
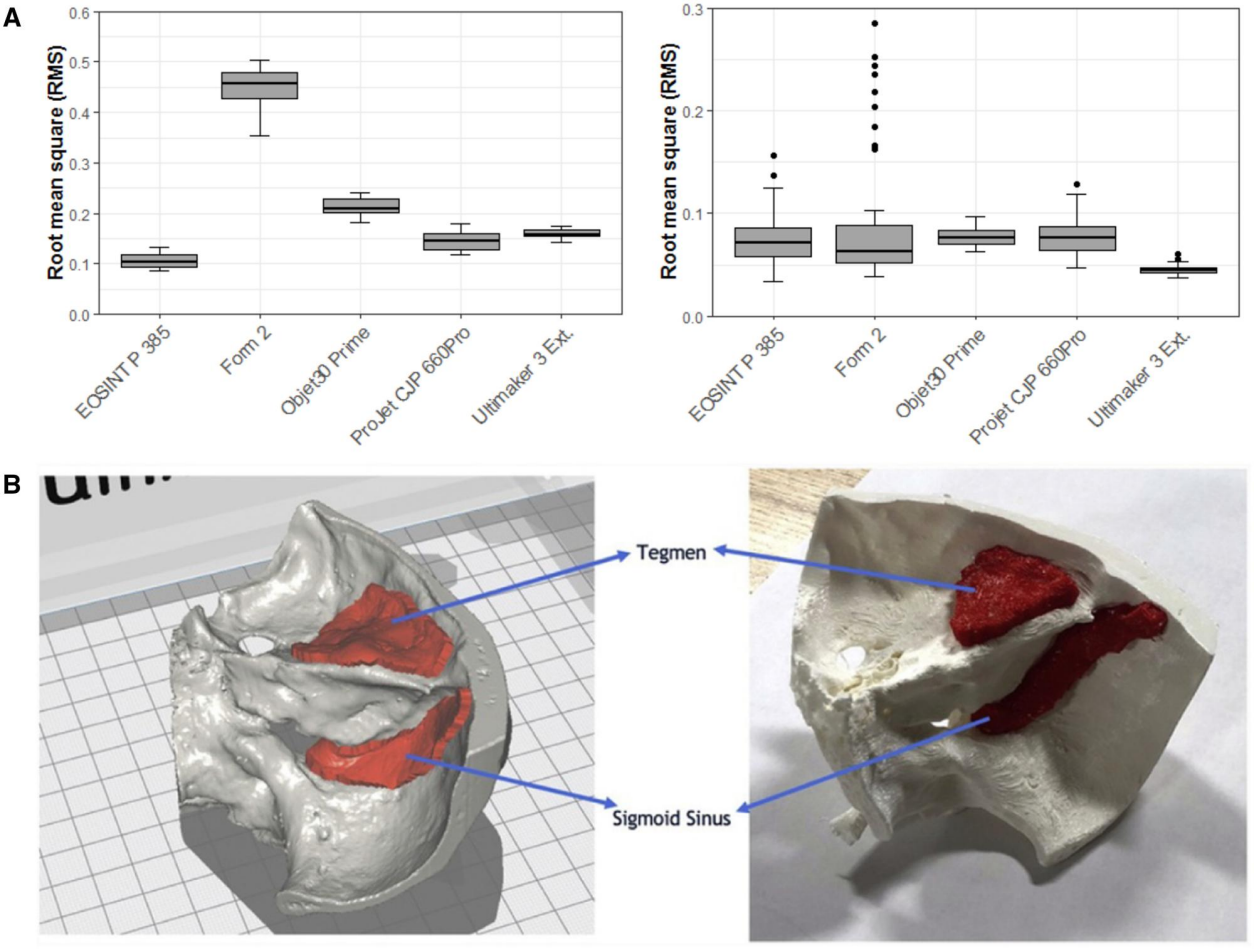
3D printed bone models. (**A**) Box plot demonstrating trueness root mean square (RMS) (mm) values and precision RMS (mm) values of models fabricated by various printers [75] (from Ref. [75] licensed under Creative Commons Attribution 4.0 license). (**B**) Computer-aided design (CAD) design of temporal bone model and the ultimate 3D-printed temporal bone model [76] (from Ref. [76] licensed under Elsevier license).

Achieving satisfying accuracy in 3D-printed bone models is of paramount importance and involves meticulous attention to various factors throughout the manufacturing process [70, 71]. Recent advancements have demonstrated that all 3D printing technologies can produce surgical models with satisfying accuracy in all three dimensions. However, certain technologies, such as material jetting (MJ) and powder bed fusion (PBF), exhibit superior accuracy compared to others like binder jetting (BJ) [72–74]. Studies comparing different printing technologies have revealed variations in dimensional accuracy but generally clinically insignificant differences (Figure 6A) [75]. Additionally, 3D-printed bone models offer the unique advantage of replicating pathological conditions, which could significantly enhance the educational experience for trainees in oral and maxillofacial surgery.

Haptic feedback, crucial for simulating tactile sensations during surgery, relies on the mechanical features of the building biomaterials [77]. Tensile strength and elastic modulus are essential parameters for achieving bone-like tactile sensations [78]. Although standardized quantitative tests for haptic feedback are lacking, a certain number of studies have proposed 3D-printed models to provide satisfactory feedback. Different printers and materials exhibit unique characteristics, influencing anatomy replication and surgical simulation. For instance, in a study by Shujaat *et al.* [79], the ProJet CJP 660Pro printer utilizing a gypsum-like material achieved the highest scores in stimulating osteotomy. However, it received lower scores for drilling holes, screw insertion and removal. Conversely, several studies have indicated that the haptic feedback of models generated from nylon-like materials was not favorable [80].

Cost remains a critical factor in the widespread adoption of 3D-printed bone models for surgical training. In-house production involves additional expenses, including software, printers, materials and operator training [74]. Material extrusion (ME) printers are often cited as cost-effective options, albeit with compromises in accuracy [81]. However, recent developments, such as a cost-effective protocol for fabricating temporal bone models using desktop printers and PLA filament, demonstrate the potential to minimize costs significantly (Figure 6B) [76]. The resulting model demonstrated anatomical accuracy (XYZ accuracy = 12.5, 12.5, 5 μm) and provided appropriate tactile feedback during surgical drilling. The total material cost for fabricating the model was approximately $1.50, significantly lower than the cost of the cadaveric temporal bone or other 3D-printed models. Additionally, printing time varies considerably depending on the technology and materials chosen, further impacting overall expenses [82]. In a study by Msallem *et al.* [75], different printing technologies, including FFF, SLA, selective laser sintering (SLS), MJ and binder jetting (BJ) were used to print anatomical mandibular models. The minimal fabrication time for each model, approximately 48 min, was achieved using SLS technology with the EOSINT P 385 (EOS GmbH, Krailling, Germany) 3D printer.

### Regenerative scaffolds and bioprinting

**Figure 7.**
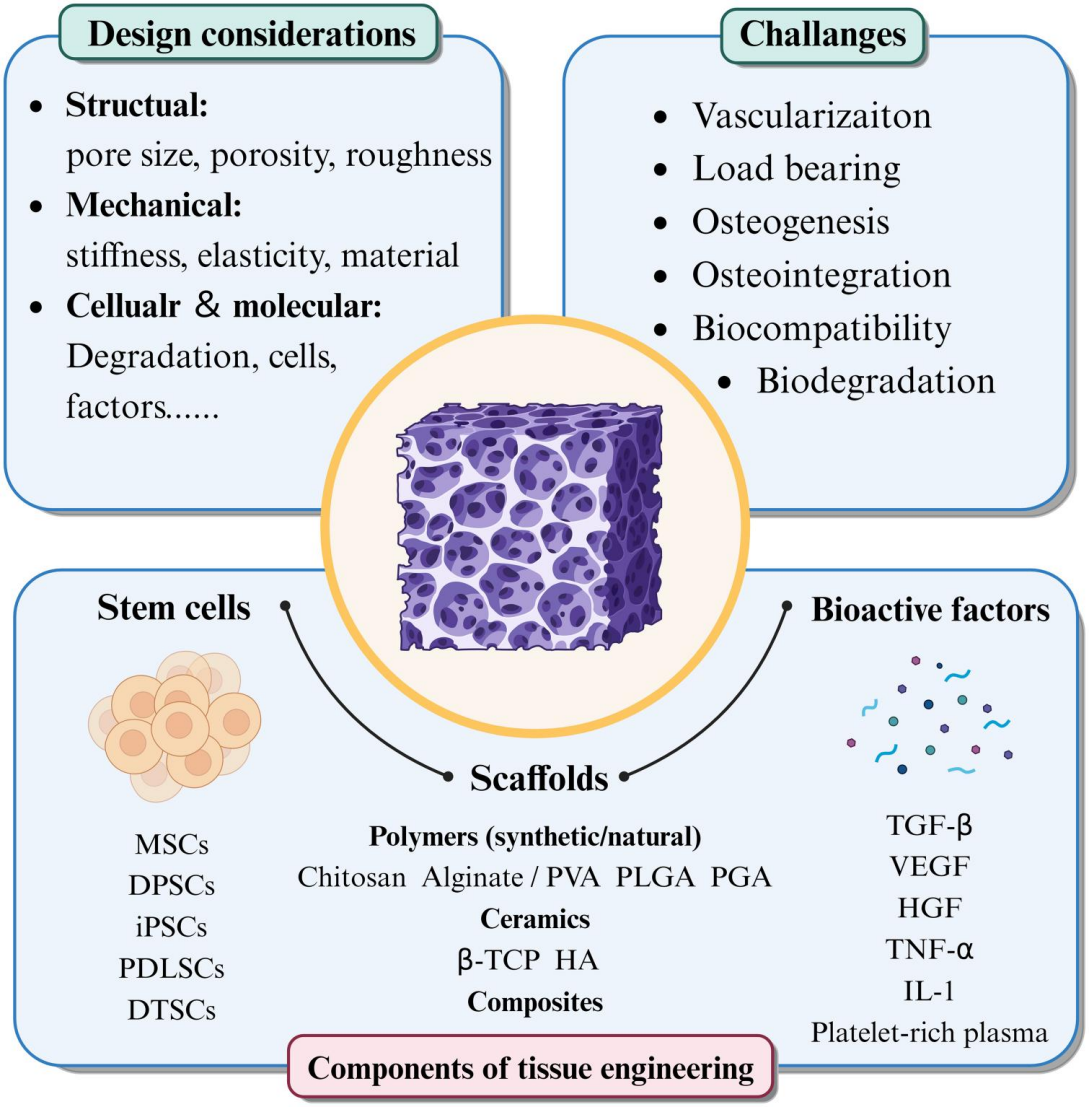
Schematic of regenerative scaffolds for tissue engineering purpose. Regenerative scaffolds generally consist of three main components including, scaffolds matrix (to support cell behaviors and tissue deposition), bioactive factors (to induce stem cell proliferation and differentiation and promote tissue regeneration and vascularization) and stem cells. Generally, we have to pay attention to several aspects when designing and developing scaffold, including mechanical, structural, cellular and molecular properties to optimize its regenerative potential. However, researchers encounter several significant challenges in tissue engineering such as osteogenesis, biodegradation and vascularization.

Traditional reconstruction strategies, such as free tissue transfer, autografts, allografts and rigid fixation, are frequently associated with limitations, including bone nonunion, donor site morbidity, restricted availability, potential disease transmission and immunogenic rejection [83–85]. Bone tissue engineering (BTE) presents a compelling alternative, offering the potential for more effective, patient-tailored solutions that promote natural tissue regeneration and functional restoration (Figure 7) [86–88]. Central to the success of BTE strategies is the concept of regenerative scaffolds [89]. These 3D frameworks serve as temporary templates, providing structural support and guidance for tissue regeneration, and the design and composition of scaffolds significantly influence their ability to mimic the native tissue environment and foster optimal healing outcomes [90, 91].

Among the various techniques available for scaffold fabrication, 3D printing has emerged as a potent tool with transformative potential, offering several extra advantages, including personalized design, high repeatability, low production costs and rapid prototyping (Table 2) [52, 100, 101]. The ability to tailor the scaffold’s architecture at a micron- and nano-scale level allows for the development of complicated architectures that greatly resemble native tissue microenvironments [102]. For example, Lee *et al.* [32] 3D printed a kagome-structure and PCL-made scaffold with excellent mechanical strength and enhanced osteoconductivity, which is a promising alternative for bone regeneration in complex and large defects. Additionally, 3D printing enables the incorporation of bioactive agents, growth factors, small molecules and multiple agent combinations directly into the scaffold matrix, further enhancing its regenerative potential [64]. For instance, the employment of growth factors, such as bone morphogenic protein-2 (BMP-2) and vascular endothelial growth factor (VEGF), can significantly improve osteogenesis and vascularization [96, 98]. Lee *et al.* [97] applied a coating of bone demineralized and decellularized extracellular matrix (bdECM) onto pre-existing 3D-printed polycaprolactone/tricalcium phosphate (PCL/TCP) structures to augment their osteoinductivity and osteoconductivity. Moreover, the incorporation of cells into these scaffolds enhances their regenerative potential and accelerates tissue healing processes, such as adipose-derived mesenchymal stem cells (ADSCs) and bone marrow-derived mesenchymal stem cells (BMSCs) [103]. Furthermore, the scalability and reproducibility of 3D printing technology facilitate its integration into clinical practice, offering clinicians a versatile tool for addressing diverse patient needs [104, 105].

**Table 2.** Regenerative scaffolds in oral and maxillofacial regions

Composition	Bioactive component	3D printing technology/equipment	Measurement	Feature	Ref
PCL	—	PED/Lab-made precision 3D printer	*In vitro* and *in vivo* (New Zealand white rabbits)	Kagome-structure Excellent mechanical robustness and enhanced osteoconductivity	[32]
Gelatin/genipin	—	3DF/3D Bioplotter (EnvisionTEC, Gladbeck, Germany)	*In vitro*	Supported the viability, attachment, and chondrogenic differentiation of hBMSC	[92]
PLA	BMP-2 coating	FDM/3DXP—One	*In vitro* and *in vivo* (mature female minipigs)	Large bone defects (12 cm^3^) reconstruction. Biomimetic coating: controlled delivery of BMP-2 (promoting bone regeneration).	[93]
Titanium	—	SLM/concept laser (Concept Laser GmbH, Germany)	*In vivo* (beagles and three patients)	In beagles: integration of broken ends in segmental bone defects after 18 months. In patients: reconstruction in large segmental mandibular defects with no mechanical complications.	[94]
Mg/nCSi	—	DLP/facility supplied by 10 Dimensions Technology Co., China	*In vitro* and *in vivo* (male New Zealand white rabbits)	Alveolar bone regeneration Good biocompatibility and osteogenic capability in *vivo*.	[95]
Silk/HAP	BMP2, VEGF and NGF	Paste extrusion/lab made	*In vitro*	Synergistic effects of BMP-2, VEGF and NGF in inducing osteoblastic differentiation *in vitro* for tissue engineering.	[96]
PCL/LAP	—	3DF/3D Bioplotter (Envisiontec, Germany)	*In vitro* and *in vivo* (Sprague Dawley rats)	Biosafety, osteoconductivity, and osteoinductivity. Vascularized bone tissue engineering in calvarial defects *in vivo*.	[64]
PLGA/TCP	rhBMP-2	MJP/Project 3510 HD Plus (3D System, USA)	*In vitro* and *in vivo* (male rhesus monkeys)	Shape retention and mineralization *in vitro*. Large compound mandibular defects regeneration and vascularization *in vivo*.	[86]
PCL/TCP	bdECM and ADSCs	Fused deposition/NM	*In vivo* (beagles)	Additional ADSC injection improved mandibular ossification in and around scaffold.	[97]
PCL/TCP scaffold + Me-HA/Me-Gel hydrogel	Resveratrol (RSV) and Strontium ranelate (SrRn)	3DF/3D-Bioplotter^®^ (Manufacturer Series, EnvisionTEC; Dearborn, MI)	*In vitro* and *in vivo* (Sprague Dawley rats)	Enhanced angiogenesis and MSC osteogenic differentiation *in vitro* Significant i*n vivo* mandibular bone formation after 8 weeks.	[98]
Ti_3_C_2_MXene/BCP/SA	BBR	Bio-Architect^®^-Pro 3D printer (Regenovo Biotechnology Co. Ltd, China)	*In vitro* and *In vivo* (New Zealand white rabbits)	Photothermal capacity and precise control of BBR release. Outstanding antibacterial and osteogenic properties *in vitro* and *in vivo.*	[99]

Ceramics and polymers represent two prominent classes of materials utilized in the fabrication of 3D-printed scaffolds for oral and maxillofacial reconstruction [106]. Ceramic-based scaffolds, such as hydroxyapatite and TCP, exhibit excellent osteoconductivity and biocompatibility, making them ideal candidates for promoting bone regeneration [107]. Polymers, on the other hand, offer versatility in scaffold design and mechanical properties, allowing for tailored solutions that accommodate specific patient needs [32, 108, 109]. Furthermore, the integration of bioresorbable polymers facilitates the gradual replacement of the scaffold with regenerated tissue over time, promoting long-term functional outcomes [110]. Composite scaffolds offer a solution by combining the strengths of individual materials while mitigating their respective weaknesses [111]. This synergistic approach allows for the design of scaffolds with enhanced mechanical properties, enhanced bioactivity and improved degradation kinetics, thereby overcoming many of the challenges associated with single material-based scaffolds [112]. For example, the incorporation of bioactive ceramics, such as calcium phosphates (CaP) and hydroxyapatite (HA), into a polymer matrix can enhance osteoconductivity and promote bone regeneration [96, 113]. Jeong *et al.* [52] fabricated a PCL/β-TCP scaffold for complex zygomatico-maxillary reconstruction.

Zhang *et al.* [98] fabricated scaffolds through 3D printing, comprising a mixture of polycaprolactone/β-tricalcium phosphate (PCL/TCP) and a hydrogel-based bioink encapsulating resveratrol and strontium ranelate. The resulting 3D-printed scaffolds, incorporating small molecules within the hydrogel, notably enhanced bone formation upon implantation within critical-sized mandibular bone defects in rat models. Bioactive metallic materials have been extensively employed in bone tissue engineering (BTE), and incorporating them into the synthetic scaffold presents a simple method to improve the osteogenesis and mechanical characteristics of composite scaffolds [114]. For instance, Wang *et al.* [115] developed PCL/Zn scaffolds with improved mechanical properties, cytocompatibility and osteogenic effect.

While still in its initial stages, bioprinting enables the printing of living tissues or organs based on cell-laden bioinks [116, 117]. These bioinks are formulated to maintain cell viability, structural integrity and functionality throughout the entire printing procedure [118]. A critical challenge lies in enabling the fabrication of finely structured three-dimensional objects while preserving the high viability and functionality of the loaded cells [119]. A wide range of bioprinting technologies has been explored for biomedical applications, with extrusion-based as one of the most promising strategies [120].

## Defect reconstruction in OMFs with great esthetic outcomes

Severe bone defects in the oral-maxillofacial region resulting from trauma and tumors not only significantly impact patients’ physiological and mental well-being but also present substantial challenges for reconstruction. According to one recent epidemiology study carried out in China, mandible fractures (31.97%) were the most common, followed by zygoma fractures (25.3%) [121]. Orbital fractures, common midface injuries, can lead to severe functional impairment [14]. The complex, anatomical region and limited intraoperative visibility pose challenges for orbital floor reconstruction. Titanium meshes have emerged as practical solutions for orbital reconstruction, and pre-bending these meshes according to 3D-printed anatomical models can achieve more exact reconstruction in terms of orbital volume [122–124]. Compared to freehand surgery, 3D virtual surgical planning plus 3D printing (VSP)-assisted surgery procedure results in significantly smaller alterations in orbit height and volume during maxillary reconstruction [125]. In the VSP group, esthetic evaluation employing color-gradient maps reveals a smoother and more symmetric curve in the post-operative appearance. Another study [126] examined the effectiveness of intraoperative bending of titanium mesh compared to pre-bent ‘hybrid’ patient-specific titanium mesh (Figure 5B). The utilization of pre-formed plates according to 3D-printed anatomical models yielded a non-significant absolute volume difference in the intervention group, while a significant difference in volume was noted in the conventional group. Moreover, surgery time was significantly reduced. An alternative methodology involves mirroring intact anatomy on the opposite side to replace the fractured orbit, thereby generating PSIs [20, 126]. Typically, the workflow for reconstructing PSIs (orbital, zygomatic, etc.) consists of five stages: (i) CT data acquisition: obtaining patient CT images. (ii) 3D model generation: creating a 3D model via computer programs (i.e. mimics) from the obtained CT images. (iii) Implant design: constructing of PSIs using the mirror reconstruction method, which involves defining a midsagittal plane as the cutting plane and mirroring the defect-free half to the defective half to maintain symmetry, followed by converting the designed implants into standard triangle language (STL) file format [18, 20]. (iv) Material selection: choosing suitable materials, such as titanium, PEEK or Ti6Al4V for specific implant construction [127, 128]. (v) 3D printing of implants.

Surgical reconstruction following total maxillectomy poses significant challenges, with conventional palatal obturator prostheses (PAPs) often failing to achieve satisfactory anatomical and functional outcomes. Gueutier *et al.* [21] introduced a reconstruction approach following total bilateral maxillectomy, which involves the implantation of a pre-developed titanium implant obtained through 3D printing. This approach yielded successful functional and anatomical outcomes without signs of rejection or infection during a follow-up period of 6–12 months, offering a new therapeutic option when free flaps are contraindicated. The vascularized fibula flap is now regarded as the gold standard in routine mandibular reconstruction. 3D/VSP has shown the potential to reduce the occurrence of radiographic non-union and complications in mandibular free fibula flap (FFF) reconstruction procedures [129]. Pu *et al.* [17] proposed a novel fibula malleolus cap to counteract these challenges during fibula flap harvesting, which significantly reduced deviations in the locations and angles of distal fibula osteotomies (Figure 4A). The utilization of the malleolus cap during simultaneous dental implant placement into flaps improved accuracy in terms of implant platform locations, apex locations, and angles [35, 130]. Compared to conventional osteotomy tools, 3D-printed guides offer universality, reusability and cost-efficiency. In cases of long-term segmental mandibular defects, mandibular deviation, malocclusion and departure from the design of the mandibular movement often occur. Dental rehabilitation and mandibular reconstruction are extensive projects requiring collaboration and subject to various influences. Li *et al.* [131] demonstrated that the integration of 3D virtual surgical planning (VSP), 3D-printed surgical guides, vascularized flap, immediate dental implants and occlusal reconstruction could offer patients enhanced appearance and occlusal reconstruction, while also reducing the necessity for multiple surgeries. Another study [132] investigated the accuracy and its influencing factors in mandibular reconstruction utilizing VSP, 3D-printed osteotomy guides and pre-bent reconstruction plates (VSP/3D-printed-guide/plate). it revealed that decreased accuracy was strongly associated with the increasing number of donor-bone segments and the length of donor-bone. These results further confirm VSP/3D-printed-guide/plate as a dependable and precise method for mandibular reconstruction.

Complete restoration of mandibular defects typically necessitates both mandibular reconstruction and dental implant placement, conducted in two sequential surgeries [133]. However, Miljanovic *et al.* [39] proposed a potential solution to streamline this process. They introduced a solution involving prepositioned dental implants, thereby reducing the need for two separate surgeries (Figure 4B). In this approach, a designed mandibular implant is placed before mandibular reconstruction with a 3D-printed guide. The strength and stability of the surgical guide were via finite element analysis (FEA), demonstrating its ability to withstand the forces encountered during surgery [134].

## Accuracy management in orthognathic surgery

**Figure 8.**
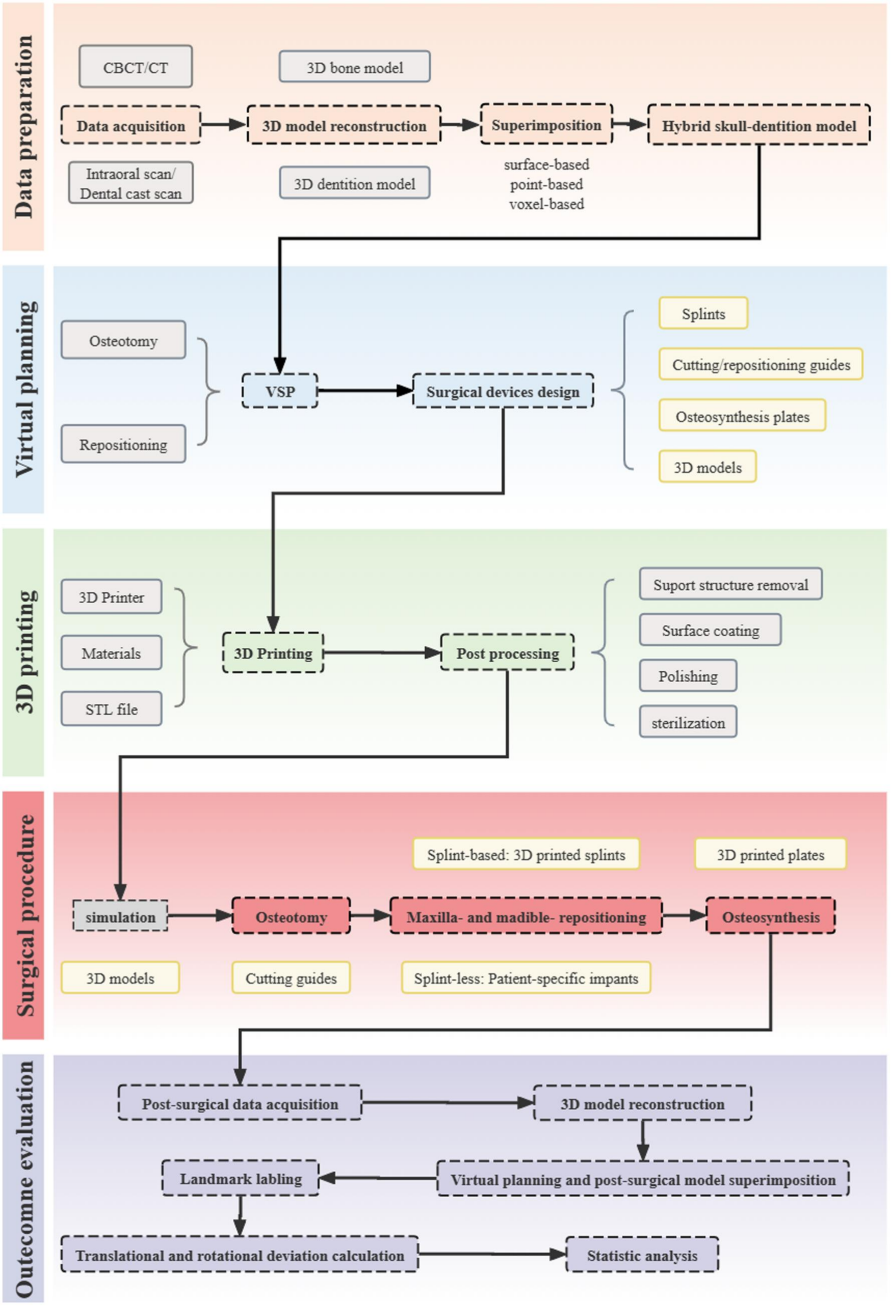
Workflow of orthognathic surgery integrated with 3D printing and virtual surgical planning (VSP). General workflow encompasses steps including data preparation, virtual planning 3D printing, surgical procedures and outcome evaluation.

Orthognathic surgery is a procedure aimed at enhancing facial aesthetics and correcting jaw bone deformities resulting from diseases, injuries or genetic factors [135]. Technological advancements have significantly affected the evolution of orthognathic surgery over the past decades, driven by the need for accurate positioning and osteosynthesis of bone segments. Virtual surgical planning enables preoperative digital design and simulation, while 3D printing facilitates accurately transferring virtual plans to the operating room [25, 136]. We have outlined the complete workflow of orthognathic surgery (Figure 8). Despite the critical importance of accurately delivering the preoperative plan to the operating room in digital orthognathic surgery, there remains no universally agreed-upon methodology or statistical method for evaluating deviations. Existing methods include calculating translational and rotational deviation between manually set landmarks on the simulation and post-surgical digital models. Some software solutions available on the market offer automated superimposition of preoperative plans and postoperative images, the establishment of *x*, *y* and *z* axes, and subsequent calculation of translational (linear) and rotational (angular) discrepancies, such as Geomagic [15] and IPS Case Designer [137].

**Figure 9.**
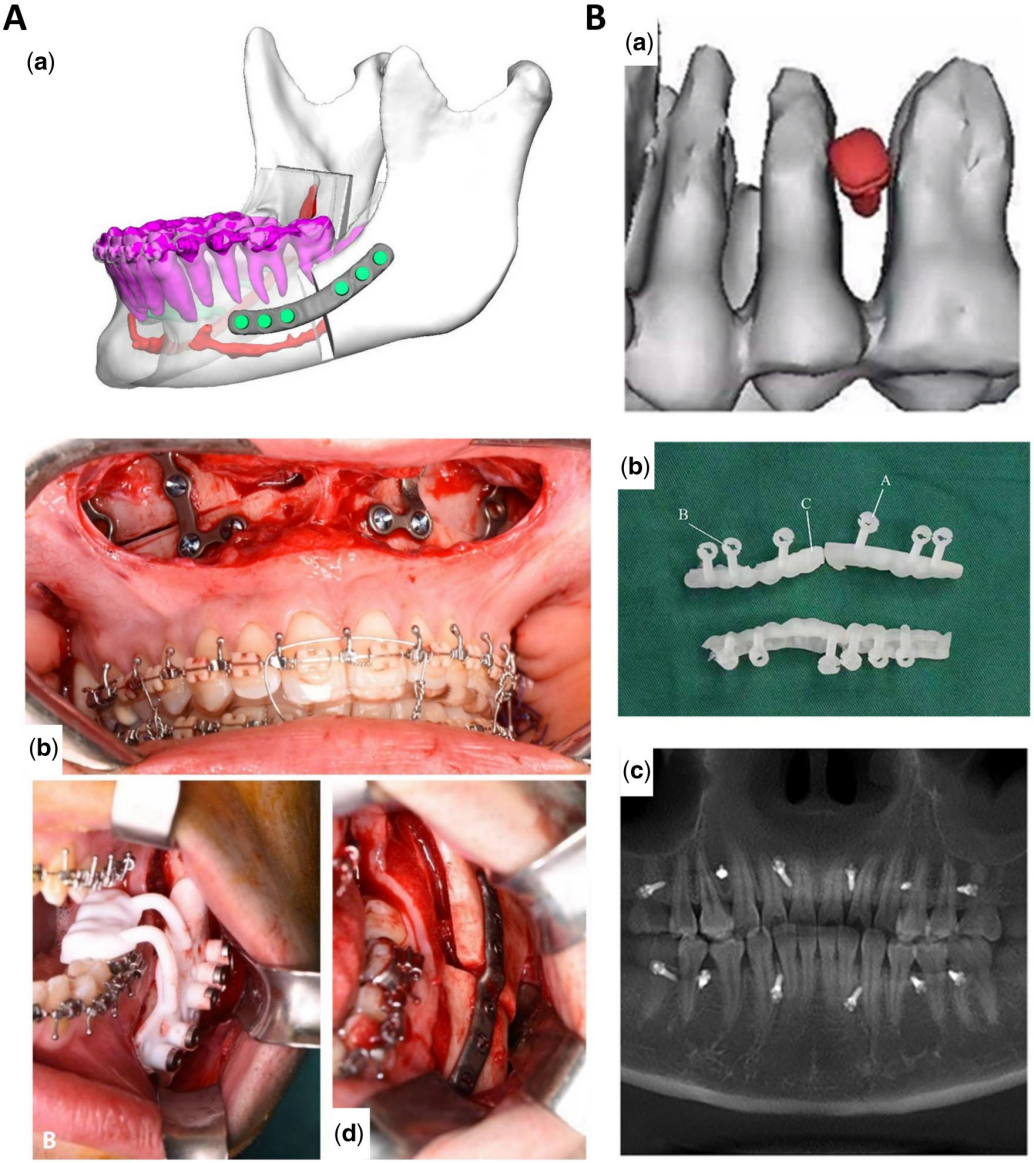
3D printed plates and screws in orthognathic surgery. (**A**) (a) Patient-specific plates CAD design; (b) intraoperative view of free hand bent plates; (c) 3D printing positioning guide; (d) CAD/CAM plate [147] (from Ref. [147] licensed under Creative Commons Attribution 4.0 license). **(**B) (a) Virtual placement of intermaxillary fixation screws; (b) digital guide model; (c) the tomographic image of IMFS installed with a digital guide [152] (from Ref. [152] licensed under Elsevier license).

Digital splints already exhibit superior accuracy, yet splints-based surgery modalities possess intrinsic errors due to the translation and rotation of the TMJ in the supine and anesthetized patient. Therefore, waferless patient-specific cutting guides and plate systems have garnered increasing interest among researchers [138, 139]. Numerous studies have demonstrated that patient-specific cutting guides and plates showed greater accuracy in repositioning maxilla compared to standard computer-aided design/computer-aided manufacturing (CAD/CAM) fabricated occlusal splints [137, 140–144]. Recently, Diaconu *et al.* [145] conducted a stratified meta-analysis including 115 single-piece Le Fort I studies utilizing PSIs, revealing that PSIs were statistically more accurate than conventional CAD/CAM splint or wafer-based osteosynthesis. Though the results are promising, studies evaluating the accuracy of PSI in orthognathic surgery are quite monotonous, as they concentrated on maxilla single-piece Le Fort osteotomies, making it challenging to conclude their potential in multisegmented maxillary osteotomies, mandibular repositioning and other areas. In mandibular orthognathic surgery, the utilization of 3D-printed PSI is less reported, as obtaining a stable reference for fixation on the lateral mandibular aspect poses significant challenges [146–148]. Compared to the maxilla, mandible repositioning deviations in PSI-aided surgery are spotted greater, but all below the 2-mm threshold [149]. These results may be attributed to inadequate hole positioning accuracy, the impact of attached masticatory muscles, and potential pterygoid-masseteric spasm [146, 150]. Although the maxilla-first approach is more common in two-jaw surgery, the argument between the maxilla-first and mandible-first approach has been lasting for decades [151]. Badiali *et al.* [147, 148] suggested that the mandibular PSI-guided mandible-first procedure accurately transfers the virtual plan to the patient (Figure 9A). This integrated procedure successfully replicated the virtually planned maxilla-mandibular positioning, allowing for precise and flexible intraoperative vertical correction. Importantly, this approach did not significantly impact frontal symmetry, achieving satisfactory aesthetic outcomes. Intraoperatively, manipulating multiple segments to place them into the PSI is technically challenging. This is due to the need to immobilize relatively small bone segments into the PSI plate while achieving movement of the individual segments in multiple planes, which may result in mutual interference between the bones [77, 150, 153, 154]. To address this challenge, Wang *et al.* [155] proposed the advantage of using PSI add-on wafers, which can improve the accuracy of surgical procedures based on virtual plans while facilitating intraoperative manipulation.

While preliminary research has confirmed the accuracy of patient-specific osteosynthesis, there remains a gap in our understanding regarding the stability of bony segments when applying this approach, with only one study evaluating 3D printing-based patient-specific osteosynthesis [15, 156]. This study revealed that 3D-printed patient-specific Ti6Al4V ELI plates processed by SLM, together with an osteotomy guide, exhibited superior accuracy in both translations and rotations compared to direct bending of the ready-made plates [15]. In addition, they measured the stability of the patient-specific plates and osteotomy guides over a 6-month period, which showed a significant improvement in stability. During the follow-up period, no major complications such as tooth loss, nerve damage, malocclusion or postoperative recurrence and infection were noted except in one patient. Intermaxillary fixation screws (IMFS) are commonly used to achieve temporary fixation of the maxilla-mandible in patients with maxillomandibular fractures or those undergoing orthognathic surgery. Cui *et al.* [152] designed and manufactured a 3D-printed guide with reduced the incidence of damage to the periodontal ligament (PDL) and tooth roots, thereby improving the success rate of IMFS implantation (Figure 9B).

## Temporomandibular joint treatment

Severe TMJ diseases, including TMJ ankylosis, end-stage TMJ osteoarthritis, comminuted condylar fractures and tumors, often necessitate the removal of the affected joint structures and subsequent reconstruction to restore both the anatomical integrity and functional capabilities [157–159]. Total alloplastic TMJ prosthesis is recognized as a reconstructive method for joint defects, and 3D printing plays a pivotal role in the manufacturing process of these complex prostheses for total TMJ replacement [160, 161]. 3D printing enables customizing TMJ prostheses according to the patient’s unique anatomical requirements [50].

**Figure 10.**
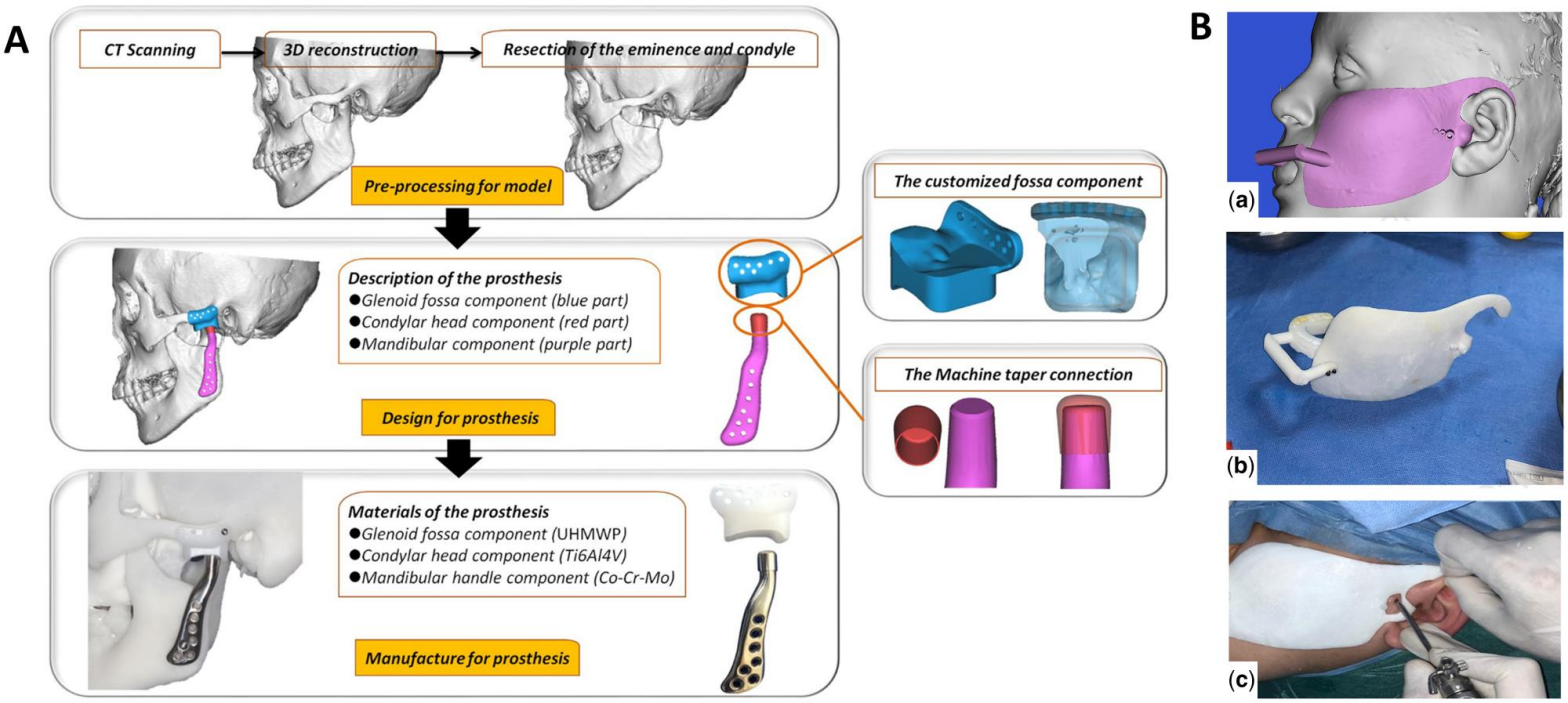
3D-printed new TMJ prosthesis with three different components. (**A**) The processing of the new TMJ prosthesis with three main components [162] (from Ref. [162] licensed under Creative Commons Attribution 4.0 license). (**B**) (a) Virtual design of the injection guide; (b) 3D-printed patient-specific guide; (c) guide fitting to the soft tissue of patients [163] (from Ref. [163] licensed under Elsevier license).

Several commercially available TMJ prostheses have been developed for clinical use, however, these prostheses do not always accurately match the unique TMJ anatomy of Chinese patients. This highlights the pressing need for research and development of TMJ prostheses designed specifically for the Chinese population. Recognizing this need, Zheng *et al.* [162] devised a novel customized TMJ prosthesis utilizing 3D printing technology (Figure 10A). The prosthesis consists of three components: the fossa component, fabricated from ultrahigh-molecular-weight polyethylene; the condylar head component, manufactured from cobalt-chromium-molybdenum alloy; and the mandibular component, fabricated from titanium alloy. Post-surgical outcomes were evaluated, and no complications were reported. After TMJ prosthesis implantation, patients showed significant improvement in pain level, jaw function, diet and maximum intermaxillary opening. During functional activities, the TMJ joint undergoes complex movements involving rotation and translation in three-dimensional space [164]. Designing a TMJ prosthesis that accurately replicates such complex kinematics presents a significant challenge. Addressing this issue, Cheng *et al.* [165] developed a 3D-printed porous condylar prosthesis using Ti-6Al-4V alloy, which possessed lower weight and a similar modulus of elasticity close to that of cortical bone. Finite element analyses (FEAs) confirmed the superior mechanical behavior of this prosthesis. The resection of the condyle often results in the detachment of the lateral pterygoid muscle, which is crucial for mandibular laterotrusion and protrusion movements. To address this concern, Zou *et al.* [166] introduced a 3D-printed titanium (Ti) alloy TMJ prosthesis, exhibiting the potential for muscle attachment and ingrowth, and potentially improving mandibular movement. Furthermore, they demonstrated the benefits of a 3D-printed porous tantalum (Ta) scaffold for facilitating muscle attachment, further augmenting the potential for clinical application [167]. The utilization of 3D-printed guides during condyle reconstruction has also proven advantageous in preventing post-surgical deviations in the condylar position [168, 169].

The TMJ constitutes a complex structure where interacting bones are covered with cartilage and separated by a small disc, facilitating smooth movement. However, the regenerative capacity of the TMJ disc is limited, posing a persistent challenge for its replacement [170, 171]. Hydrogels are characterized by a three-dimensional porous network structure containing a large amount of water, which provides lubricating properties for joints. However, its mechanical properties, such as low strength and toughness, hinder its application as an artificial weight-bearing TMJ disc [116, 172, 173]. To address this limitation, Jiang *et al.* [174] synthesized a novel TMJ disc by combining polyvinyl alcohol (PVA) hydrogel with a 3D-printed polycaprolactone (PCL) framework.

PCL implants were manufactured using a layer-by-layer deposition technique, while hydrogels are cross-linked by a simple cyclic freeze-thaw method. The mechanical strength of the resulting PVA + PCL artificial intervertebral discs has comparable mechanical strength to natural intervertebral discs. Experiments with the artificial disc in goats with TMJ disc defects demonstrated that it was able to maintain joint stability and protect the condylar cartilage and bone from damage. The PCL implants were fabricated using a layer-by-layer deposition technique, with the hydrogel crosslinked through a cyclic freeze-thaw method. Compared to the natural disc, the resulting PVA + PCL artificial disc exhibited comparable mechanical robustness. Experimental implementation of the artificial disc in goats demonstrated its ability to preserve the stability of joints and protect the condylar structures from damage. Combining 3D-printed scaffolds and mesenchymal stem cells, tissue engineering presents another promising alternative for TMJ repair [175, 176]. This approach involves fabricating scaffolds with precise dimensions and structures using 3D printing technology and incorporating mesenchymal stem cells to promote tissue regeneration. The combination of these innovative techniques holds great potential for overcoming the limitations of current treatment options and enhancing the prospects for successful TMJ repair [177].

Some therapies for TMJ disorders involve injecting substances into specific compartments of the joint, such as the superior or inferior compartments. To minimize the risk of complications, such as bleeding, hematoma and intracranial perforation, researchers have explored using 3D-printed surgical guides for precise needle insertion (Figure 10B) [163, 178]. These guides aim to ensure accurate placement and reduce the likelihood of adverse events. The findings of these studies have demonstrated minimal angle deviation, indicating the potential of 3D-printed guides to enhance the safety and precision of needle insertion procedures. Furthermore, in cases of post-traumatic TMJ ankylosis, a tissue graft is inserted to restore joint function during inter-positional arthroplasty, 3D-printed splints have shown promising utility in occlusal stabilization and maintaining the desired gap between the graft and adjacent structures [179].

## Other applications

There is a growing trend in using 3D-printed models in medical education, especially in anatomy teaching and surgical training [180–182]. A series of randomized control trials and meta-analysis have reported that compared with traditional educational materials such as 2D CT images and illustrations, 3D-printed skull models are inexpensive, accurate and fast to produce teaching material in morphology education [183–186]. For instance, the cost of printing each model in ABS using a FDM printer is generally less than $10. [187]. Moreover, 3D-printed models can provide surgeons with a tangible platform providing visual and haptic perceptibility for meticulous dissection and reconstruction exercises, allowing them to familiarize themselves with the complexities of specific pathologies before entering the operating room [73, 188].

Recently, it has been gradually evident that ultrasound imaging can also serve as base data to generate 3D models using surface-rendered sonographic views. As a result, researchers have dedicated their great effort to generating 3D models from prenatal surface-rendered views and demonstrated their potential in parental education of fetal malformations [189–191]. For instance, Nicot *et al.* [189, 190] produced ABS models of cleft lip fetus using a low-cost 3D printer and a surface-rendered oropalatal (SROP) sonographic view. Those models necessitate 60 70 g of ABS and a printing time of about 6∼7 h, costing only France $2 [189–191]. Such 3D models could be extended to replicate other facial malformation replications and enhance parents’ comprehension of the deformities of their unborn children.

Suspicious radiological images of jaw bone often necessitate histopathological examination to confirm a final diagnosis. The employment of guided biopsy using 3D-printed guides could minimize the chance of devitalization of the neighboring teeth and nerves [192, 193]. Valdec *et al.* [194] designed and 3D-printed a tooth-supported drilling template trephine biopsy, demonstrating high accuracy and great predictability sampling, while possessing minimal invasiveness. Compared to free-handed biopsies, guided procedures showed significantly lower mean deviation between the biopsy axes [38].

Some patients may experience reoccurrence after surgical resection, making localized anti-tumor therapy crucial for inhibiting local recurrence [195, 196]. With the development of biomaterials and drug delivery systems, 3D porous scaffolds capable of controlled anti-tumor drug release have garnered increasing interest in cancer treatment [197–199]. Local chemotherapy using chemo drug-loaded scaffolds can significantly reduce the side effects of chemo drugs on non-tumorous tissues and organs [200, 201]. More recently, stimuli-responsive scaffolds that enable on-demand drug release in the presence of external stimuli have gained increasing interest [202, 203]. For instance, near-infrared (NIR)-responsive drug-loaded scaffolds are among the most extensively studied systems for cancer therapy, as they can achieve NIR-induced drug release and thermal effect simultaneously, exerting synergistic chemo-photothermal anti-cancer effects [204]. Dutta *et al.* [205] 3D-printed hydrogel scaffolds with NIR-controlled release of antitumor drugs, effectively killing osteosarcoma cells.

## Conclusion and future outlook

The integration of 3D printing technology has significantly transformed oral and maxillofacial surgery by enabling customization and personalization through the creation of 3D-printed surgical devices including PSIs, surgical guides, splints and 3D models. Furthermore, the convergence between 3D printing and virtual surgical planning has revolutionized surgical workflows, leading to improved accuracy, reduced surgical time, and decreased costs. Consequently, these advancements have greatly benefited procedures such as bone reconstruction, orthognathic surgery, and TMJ treatment, while also enhancing patient communication and education. Overall, 3D printing and its associated surgical devices have streamlined workflow and elevated precision across various applications within oral and maxillofacial surgery.

However, there are still some research limitations of 3D printing during oral and maxillofacial surgical operations. Firstly, the diversity of clinically available building materials is limited and properties haven’t been optimized enough. Take titanium for example, osseointegration and stress shielding are the two biggest concerns needing improvement. Optimization of internal structures and proper surface modifications are needed in further research, together with new building material exploitation. Besides, confirmation of long-term implantation stability of biomaterials is absent in most studies, leading to possible worries. Additionally, the cost-effectiveness of 3D printing has to be carefully managed for broader clinical applications, which is greatly impacted by technology and its associated materials. Besides, the initial investment required for acquiring 3D printers and the ongoing expenses for materials can be significant and there may be a learning curve for surgeons and healthcare professionals to become proficient in utilizing 3D printing technology effectively. Moreover, the regulatory landscape surrounding 3D-printed medical devices is still evolving. Standardized evaluation methodology to ensure the safety and efficacy of 3D-printed implants and other surgical tools hasn’t been cautiously discussed and established so far, which is the most crucial aspect regrading 3D printing applications. Regulatory authorities must establish guidelines and standards for producing and using 3D-printed medical devices to ensure patient safety.

Encouragingly, there are several exciting future directions to further exploit the potential of 3D printing in oral and maxillofacial surgery. With the development in regenerative medicine, 3D printing of bioactive scaffolds holds the potential to generate live tissues similar to the original ones. These scaffolds can be 3D-printed with biocompatible materials and designed to mimic the structure and properties of natural tissues. By incorporating factors that promote tissue growth and vascularization, these scaffolds can enhance the body’s ability to regenerate damaged or missing tissue following surgery. Bioprinting creates functional tissue structures by printing living cells laden with biocompatible biomaterials. In oral and maxillofacial surgery, bioprinting holds promise for the fabrication of functional tissues such as bone, cartilage, or mucosa. Drug delivery systems can be incorporated into implants or scaffolds to deliver therapeutic agents directly to the surgical site. This can include antimicrobial agents to prevent infection, growth factors to promote tissue regeneration, or analgesics to manage post-operative pain. By delivering drugs locally to the site of surgery, these systems can improve therapeutic outcomes while minimizing systemic side effects.

In conclusion, 3D printing and generated 3D-printed surgical devices have revolutionized the field of oral and maxillofacial surgery by offering customized and precise solutions for PSIs, surgical guides, and models. It enhances surgical accuracy, improves patient outcomes, and streamlines the production process. However, challenges such as cost and regulatory considerations must be addressed for the broader adoption of 3D printing in oral and maxillofacial surgery. By addressing those limitations and diving into promising research directions, 3D printing holds great promise for further advancements in innovative techniques, improved patient outcomes, and enhanced overall experiences in oral and maxillofacial surgery.

## Supplementary Material

rbae066_Supplementary_Data
