# Supplementary material for: 3D printing materials and 3D printed surgical devices in oral and maxillofacial surgery: design, workflow and effectiveness
Source: Regen Biomater. 2024 Jun 27;11:rbae066. doi: 10.1093/rb/rbae066 (PMC11338467; doi:10.1093/rb/rbae066)
Supplement: rbae066_Supplementary_Data [file rbae066_supplementary_data.docx]

**Supplementary Material**

**3D Printing Materials and 3D Printed Surgical Devices in Oral and Maxillofacial Surgery: Design, Workflow and Effectiveness**

Xiaoxiao Wang ^1,2,†^, Min Mu ^2,†^, Jiazhen Yan ^3^, Bo Han ^4^, Rui Ye ^1,^*, Gang Guo ^2,*^
^1^ State Key Laboratory of Oral Diseases & National Center for Stomatology & National
Clinical Research Center for Oral Diseases & Department of Orthodontics, West China
Hospital of Stomatology, Sichuan University, Chengdu 610041, Sichuan, China
^2^ Department of Biotherapy, State Key Laboratory of Biotherapy and Cancer Center,
West China Hospital, Sichuan University, Chengdu, 610041, China
^3^ School of Mechanical Engineering, Sichuan University, Chengdu 610065, China
^4^ School of Pharmacy, Shihezi University, and Key Laboratory of Xinjiang
Phytomedicine Resource and Utilization, Ministry of Education, Shihezi, 832002,
China
*Correspondence to: Rui Ye (yerui@scu.edu.cn); Gang Guo ([guogang@scu.edu.cn](mailto:guogang@scu.edu.cn))

^†^ These authors contributed equally to this work.

1. **Literature analysis**

We conducted a systematic search of the Medline (PubMed) database, Scopus database, and Web of Science database from January 1, 2005, to December 31, 2023, using the following search equation:

((‘‘Printing, Three-Dimensional’’[Mesh]) OR ("additive manufacturing"[Title/Abstract]) OR ("three-dimensional print*"[Title/Abstract]) OR ("3D print*"[Title/Abstract]) OR("3-dimensional print*"[Title/Abstract]) OR ("stereolithography"[Title/Abstract]) OR ("selective laser sintering"[Title/Abstract]) OR ("3D-printed model"[Title/Abstract]) OR ("patient-specific implants"[Title/Abstract]) OR ("rapid prototyping"[Title/Abstract])) **AND** (("maxill*"[Title/Abstract]) OR ("orthognathic"[Title/Abstract]) OR ("mandib*"[Title/Abstract]) OR("tempor* "[Title/Abstract]) OR("sinus augmentation"[Title/Abstract]) OR ("sinus lift"[Title/Abstract]) OR ("temporomandibular joint"[Title/Abstract]) OR ("TMJ"[Title/Abstract]) OR ("bilateral sagittal split osteotomy"[Title/Abstract]) OR ("craniofacial"[Title/Abstract]) OR ("temporo-orbital"[Title/Abstract]) OR ("craniomaxillofacial"[Title/Abstract]) OR ("zygomatic"[Title/Abstract]) OR("jaw"[Title/Abstract]) OR ("maxillectomy"[Title/Abstract]))

This search strategy only targeted articles with search terms in the title or abstract, without language restrictions. The PubMed search strategy was modified for use in the other databases, employing similar keywords. Additionally, other relevant original literature was identified through manual searches. We included all original full papers written in English that addressed the fabrication of 3D printed surgical devices (including patient-specific implants, 3D printed models, splints, 3D printed surgical guides, and regenerative scaffolds) for use in oral and maxillofacial surgery. Case reports, case series, pilot studies, and comparative studies were considered eligible for inclusion in our analysis.

To assess the geographical distribution of Investigator-Initiated Clinical Trials/Research (IIT/IIR) and Industry-Sponsored Clinical Trials (IST), we analyzed the corresponding addresses' countries. Basic research, animal studies, case reports, case series, and pilot studies were excluded from this statistical analysis.

1. **Patent analysis**

This review utilized the Espacenet database, Web of Science database, and [Patentscope database](https://patentscope.wipo.int/search/en/search.jsf) ([WIPO, World Intellectual Property Organization](https://www.wipo.int/)) for patent analysis. The selection of patents adhered to specific inclusion criteria: recent patents published between January 1, 2005, and December 31, 2023, in any language, with keywords "3D printed surgical guides" and "oral and maxillofacial surgery" appearing in the title or abstract. Subsequently, the patent contents were categorized by country to facilitate further analysis of geographical distribution.
